# Editorial: 2D-Layered Nanomaterials: Chemical Functionalization, Advanced Characterization, and Tribological Properties

**DOI:** 10.3389/fchem.2022.840213

**Published:** 2022-02-17

**Authors:** Andreas Rosenkranz, Carsten Gachot, Ali Erdemir

**Affiliations:** ^1^ Department of Chemical Engineering, Biotechnology and Materials, University of Chile, Santiago, Chile; ^2^ Department of Mechanical and Industrial Engineering, Design Engineering and Product Development, TU Wien, Vienna, Austria; ^3^ Department of Mechanical Engineering, Texas A&M University, College Station, TX, United States

**Keywords:** 2D nanomaterials, MXenes, chemical functionalization, friction, materials characerization

Nowadays, a significant amount of the global energy output is consumed to overcome friction- and wear-induced problems or even damages, a situation that urgently calls for more efficient tribological solutions and strategies (Holmberg and Erdemir, 2017; Holmberg and Erdemir, 2019; Shah et al., 2020). 2D-layered nanomaterials such as graphene, graphene oxide (GO), MoS_2_, etc., have gained substantial attention for their use as solid lubricants and lubricant additives thus serving as promising alternatives to conventional lubrication approaches under harsh operating conditions (Berman et al., 2018; Zhang et al., 2019; Rosenkranz et al., 2020). Since 2011, the class of 2D nanomaterials has been significantly expanded by the discovery of a new class of 2D transition metal carbides and/or carbonitrides (Naguib et al., 2011; Naguib et al., 2014). These newly emerging nanomaterials, with Ti_3_C_2_T_x_ as their most prominent member, are called MXenes due to their origin in MAX-phases and their structural similarity to graphene. MXene nano-sheets have been extensively studied and also used in energy storage, catalysis, and water purification (Anasori et al., 2017; Gogotsi and Anasori, 2019; Naguib et al., 2021). During the last 2 years, MXenes have experienced increasingly more attention within the tribological community due to their remarkable solid lubrication abilities and outstanding anti-wear performance (Malaki and Varma, 2020; Marian et al., 2020; Wyatt et al., 2021).

Although they present a new paradigm as promising alternatives to conventional lubrication approaches, 2D-layered nanomaterials as solid lubricants and lubricant additives have still some shortcomings that need to be overcome to further optimize their friction and wear performance (Berman et al., 2018; Zhang et al., 2019; Rosenkranz et al., 2020). Concerning solid lubricants, the adhesion strength of the deposited nanomaterials/nano-films to the substrate is critical for the resulting tribological performance (Malaki and Varma, 2020). Regarding lubricant additives, the generally hydrophilic character of these nanomaterials lowers their dispersibility in hydrophobic oils, which goes hand in hand with stability and sedimentation problems over time. Irrespective of the nanomaterial and the application (solid lubricant or lubricant additive), the surface chemistry of these nanomaterials is considered as the key factor to solve these problems. The existing surface terminations can be used to chemically functionalize the 2D-layered nanomaterials, thus allowing for enhanced adhesion strength and improved dispersibility. Chemical modifications need to be complemented by advanced, high-resolution materials characterization (chemical and structural) to ensure the success of the functionalization process. Moreover, materials characterization is of utmost importance for shedding light onto the very complex nature of tribologically involved interfaces prior to and after the tribological experiments, to understand their underlying friction and wear mechanisms.

In the framework of this special issue, there are several highly innovative manuscripts ranging from new exfoliation approaches, nanoscale humidity-dependent lubrication mechanisms to the performance of hybrid nanocomposites. In this context, Zhang et al. presented a new exfoliation method for MoS_2_, which results in nano-flakes with bigger sizes and increased reaction yields. Claerbout et al. numerically investigated the frictional properties of multilayer MoS_2_ during sliding in the presence of water by nonequilibrium molecular dynamics simulations. Zhu et al. addressed the contribution of four oxygenated acids to oxygen-containing functional groups in Hummers’ method using first principle methods. Li et al. studied the friction behavior of graphene in air and nitrogen atmosphere thus considering the microstructural evolution caused by the variation of test environments and their effects on the coefficient of coefficient. Regarding composite materials, Jakubczak et al. verified the performance of graphene-based nano-hybrids, while Chen et al. addressed the tribological performance of self-assembled multilayer films of (GO/PDDA)n.

At this point, we would like to express our sincere thanks to Frontiers Publishing Group for providing the opportunity for this special issue. We would also thank the authors of each chapter for their great contributions. Lastly, we would also thank the readers for their interest in our special issue. Undoubtedly, they will appreciate the importance of 2D materials in combatting friction and wear although there remain several more challenges to overcome for large-scale industrial applications.
